# Prolonged mitotic arrest induced by Wee1 inhibition sensitizes breast cancer cells to paclitaxel

**DOI:** 10.18632/oncotarget.17848

**Published:** 2017-05-13

**Authors:** Cody W. Lewis, Zhigang Jin, Dawn Macdonald, Wenya Wei, Xu Jing Qian, Won Shik Choi, Ruicen He, Xuejun Sun, Gordon Chan

**Affiliations:** ^1^ Department of Oncology, University of Alberta, Edmonton, Alberta, Canada T6G 1Z2; ^2^ Experimental Oncology, Cross Cancer Institute, Edmonton, Alberta, Canada T6G 1Z2; ^3^ Cancer Research Institute of Northern Alberta, University of Alberta, Edmonton, Alberta, Canada T6G 2J7

**Keywords:** cell cycle checkpoint, Wee1 kinase, paclitaxel, mitotic catastrophe, breast cancer

## Abstract

Wee1 kinase is a crucial negative regulator of Cdk1/cyclin B1 activity and is required for normal entry into and exit from mitosis. Wee1 activity can be chemically inhibited by the small molecule MK-1775, which is currently being tested in phase I/II clinical trials in combination with other anti-cancer drugs. MK-1775 promotes cancer cells to bypass the cell-cycle checkpoints and prematurely enter mitosis. In our study, we show premature mitotic cells that arise from MK-1775 treatment exhibited centromere fragmentation, a morphological feature of mitotic catastrophe that is characterized by centromeres and kinetochore proteins that co-cluster away from the condensed chromosomes. In addition to stimulating early mitotic entry, MK-1775 treatment also delayed mitotic exit. Specifically, cells treated with MK-1775 following release from G1/S or prometaphase arrested in mitosis. MK-1775 induced arrest occurred at metaphase and thus, cells required 12 times longer to transition into anaphase compared to controls. Consistent with an arrest in mitosis, MK-1775 treated prometaphase cells maintained high cyclin B1 and low phospho-tyrosine 15 Cdk1. Importantly, MK-1775 induced mitotic arrest resulted in cell death regardless the of cell-cycle phase prior to treatment suggesting that Wee1 inhibitors are also anti-mitotic agents. We found that paclitaxel enhances MK-1775 mediated cell killing. HeLa and different breast cancer cell lines (T-47D, MCF7, MDA-MB-468 and MDA-MB-231) treated with different concentrations of MK-1775 and low dose paclitaxel exhibited reduced cell survival compared to mono-treatments. Our data highlight a new potential strategy for enhancing MK-1775 mediated cell killing in breast cancer cells.

## INTRODUCTION

Cyclin dependent kinase (Cdk)-1/cyclin B1 is the key complex, that when active, initiates mitosis with subsequent inactivation triggering mitotic exit [1]. Cdk1 activity is tightly regulated in interphase by Wee1 and Myt1 kinases, which add inhibitory phosphates to Cdk1 on threonine 14 and tyrosine 15 thus preventing premature mitosis [2, 3]. In this way, Wee1 and Myt1 kinases maintain competent G_1_/S, intra-S and G_2_/M checkpoints that ensure cells have completed DNA synthesis as well as repaired any damaged DNA prior to mitosis [4]. When mitosis begins, the inhibitory phosphates on Cdk1 are removed by Cdc25 phosphatases [3] and Wee1 and Myt1 are subsequently inactivated (and degraded) [5]. However, a small amount of inactive Wee1 remains throughout mitosis [6–8]. At the end of mitosis, mitotic exit is characterized by the re-phosphorylation and inhibition of Cdk1 and degradation of cyclin B1 [9]. Both are dependent upon the reactivation of residual Wee1 during anaphase [6–8, 10]. In the absence of Wee1 activity, mitotic cells maintain high levels of cyclin B1 and low levels of phosphorylated tyrosine 15-Cdk1 [6, 7].

Wee1 is reported to be overexpressed in several cancers including breast [11–13]. High Wee1 activity helps reinforce the DNA damage checkpoints and facilitates DNA repair, which in turn allows cancer cells to resist genotoxic therapies [14]. Thus, Wee1 has become a target of therapeutic interest. Wee1 activity can be chemically inhibited by the small molecule inhibitor MK-1775 [15], which is undergoing phase I/II clinical trials in combination with several different genotoxic therapies including cisplatin and radiation (clinicaltrials.gov). The rationale of these clinical trials is largely supported by human cell line studies, including recent publications, which report that MK-1775 sensitizes cancers of the brain [16] and head and neck [17] to the genotoxic drug cisplatin as well as pancreatic cancer cells to gemcitabine [18].

The mechanism of MK-1775-mediated cell death in breast and other cancers has been largely attributed to either premature mitosis or entry into mitosis with damaged DNA following exposure to genotoxic agents [13, 19]. These treatment strategies induce chromosome defects that are incompatible with viable mitosis. MK-1775 treatment is shown to force HeLa and breast cancer cells into mitosis that were previously arrested in G1/S by treatment with thymidine, gemcitabine, or hydroxyurea [19]. While in mitosis these cells display defects including chromosome pulverization and abnormal microtubule organization. A similar morphology has been described in Chinese hamster ovarian (CHO) cells [20] and pancreatic cancer cells [21] that underwent mitosis with unreplicated genomes (MUG), a morphological marker of mitotic catastrophe. Mitotic catastrophe is a major mode of cell death in tumours following genotoxic treatment, however, its molecular mechanism is poorly defined [22]. In the MUG studies, cells were first treated with etoposide, gemcitabine, or thymidine to induce an S phase arrest and then treated with either caffeine (ATM/ATR inhibitor) or UCN-01 (Chk1/2 inhibitor) to bypass the S and G_2_/M checkpoints and force cells into mitosis [21]. The resulting mitotic cells exhibited centromere fragmentation due to torsional strain caused by incomplete centromeric DNA replication leading to abnormal DNA condensation resulting in a prolonged mitotic arrest [20, 21]. In our study, we will refer to the MUG morphology as centromere fragmentation. Whether inhibiting Wee1 with MK-1775 also induces centromere fragmentation has yet to be confirmed, but elucidating the role of Wee1 in this process is important in terms of understanding the molecular pathways leading to mitotic catastrophe. These observations explain in part the synergistic activity of MK-1775 with genotoxic agents in the clinic.

In addition to enhancing the efficacy of genotoxic therapeutics, there is also evidence that MK-1775 can be used as a mono-treatment, particularly in cells that lack p53 [15, 19]. This observation implies that inhibiting Wee1-mediated mitotic exit may on its own promote cell death, although the steps are not clear. In addition to inducing cell death in G1/S synchronized cells, MK-1775 also prolongs mitosis in cells with fully replicated chromosomes that are released from prometaphase as marked by high cyclin B1 levels and a mitotic specific phosphorylation on serine 10 of histone H3 [7]. Prolonging mitosis with anti-mitotic drugs such as nocodazole, paclitaxel, and the Eg5 kinesin inhibitor AZ-138 have been previously shown to induce cell death [23]. To our knowledge no study has determined the cell fate of a mitotic arrest induced by the loss of Wee1. We believe that MK-1775 disrupts the cell cycle and induces cell death by two different mechanisms, both of which cause a mitotic arrest: 1) premature mitosis in cells with under replicated DNA and 2) unregulated Cdk1 activity in prometaphase cells. We hypothesize that the key to the MK-1775 induced cell death in both cases is dependent on a prolonged mitotic arrest. We predict that the addition of other clinical agents that also induce a mitotic arrest, such as the microtubule poison paclitaxel, will enhance the induction of Wee1 mediated cell death.

MK-1775 was recently shown to enhance the efficacy of paclitaxel in HeLa cells and another microtubule poison vincristine, in leukemia cells [7]. Importantly, paclitaxel and its derivatives are a first line treatment against breast cancer [24]. However, no cell line studies or clinical trials have specifically explored the effects of MK-1775 and paclitaxel in solid tumours affecting the breast. Here we will show that breast cancer cell lines (T-47D, MDA-MB-231, MDA-MB-468 and MCF7) undergo a prolonged mitotic arrest in response to MK-1775 treatment. We also show that MK-1775 can sensitize breast cancer cells to paclitaxel treatment. Therefore, our data provide a strong rationale to explore the potential benefits of combining MK-1775 and paclitaxel in future clinical studies on breast cancer.

## RESULTS

### Inhibition of Wee1 promotes premature mitosis

**Figure 1:**
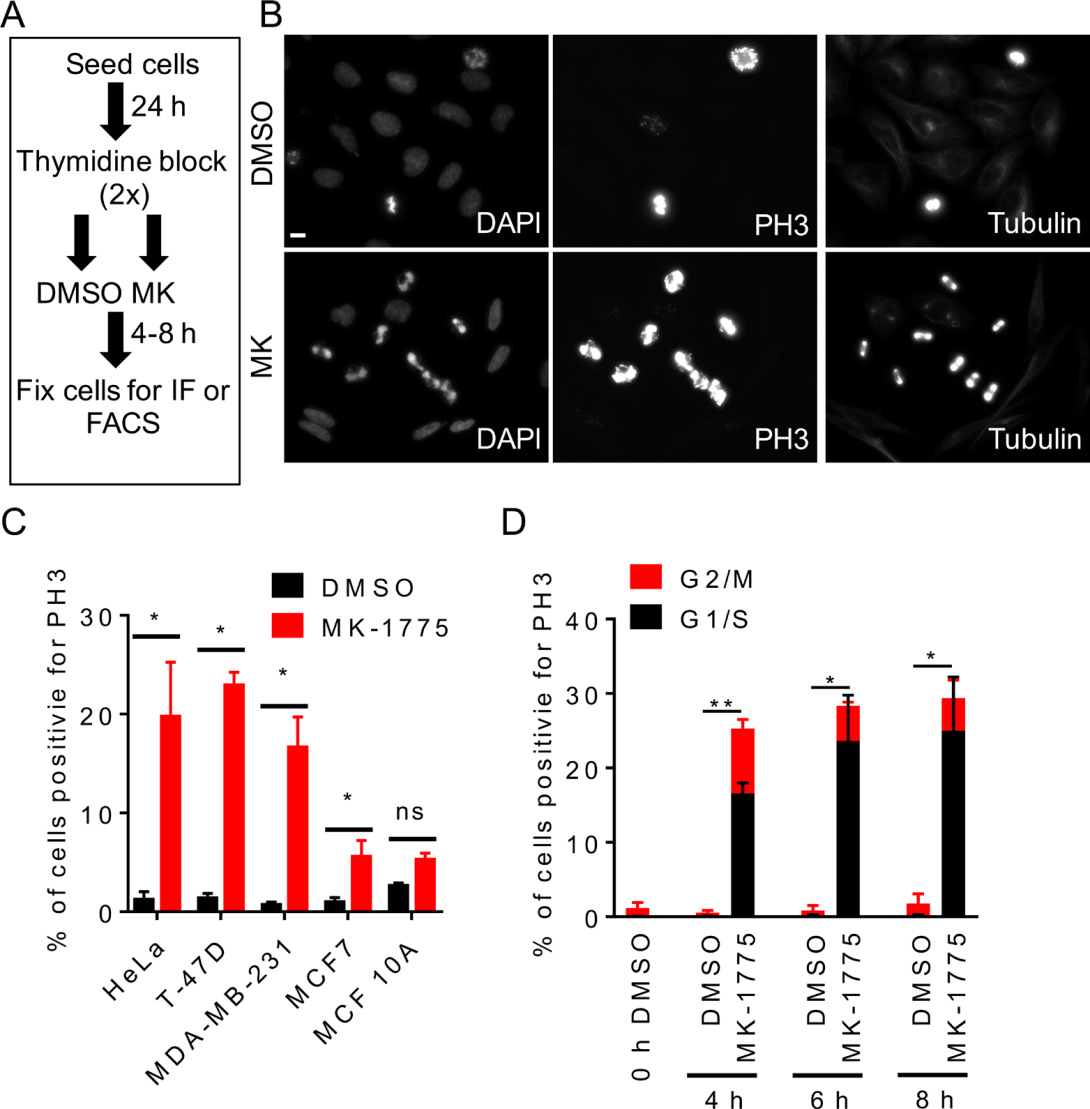
Inhibition of Wee1 kinase promotes premature entry into mitosis HeLa cells were released from G1/S phase into media containing either DMSO or MK-1775 (MK) and then fixed at indicated times. (**A**) Experimental flow chart depicting treatments and times. (**B**) Cells were stained for DNA, PH3, and microtubules and then analyzed by immunofluorescence microscopy 4 h post treatment. Scale bar = 10 µm. (**C**) Indicated cell lines were treated with DMSO or MK-1775 for 4 h and then analyzed by immunofluorescence microscopy for PH3 and DNA. Percent total cells positive for PH3 is shown. Student *t*-test was used to determine significance between DMSO and MK-1775 (*p < 0.05). (**D**) Cells stained for PH3 and DNA were analyzed by FACS to determined cell cycle phase. Average percentage of cells positive for PH3 relative to DNA staining are shown. Error bars are standard error of the mean. Black bars represent cells in the G1/S phase and red bars represent cells in the G2/M phase. Statistical significance was determined by student *t*-test (*p < 0.05 and **p < 0.005

**Figure 2:**
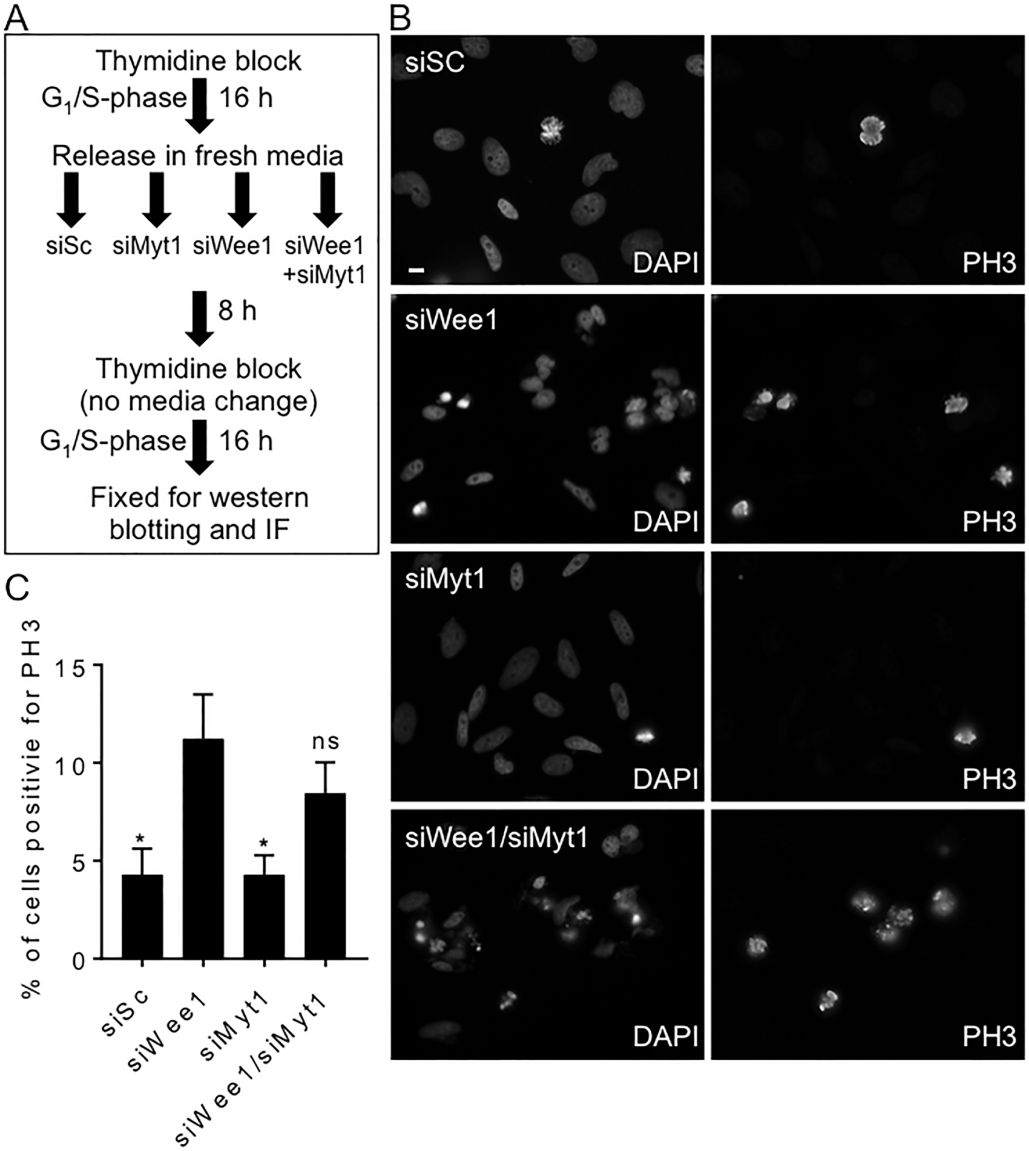
siRNA mediated knockdown of Wee1, but not Myt1, induces premature mitosis HeLa cells synchronized in G1/S phase were transfected with scrambled siRNA (siSc) and siRNAs against Wee1, Myt1, and both Wee1 and Myt1. (**A**) Experimental flow chart depicting timing and treatments. (**B**) Cells were fixed 24 h post siRNA transfection and then stained for DNA and PH3. Scale bar = 10 μm. (**C**) The average percentage of cells positive for PH3 (relative to DNA staining) are graphed. Student *t*-test was used to determine statistical significance between siWee1 and other treatments (*p < 0.05). Standard error of the mean bars are shown. Experiments were repeated at least three times.

To examine the role of Wee1 kinase in preventing premature mitosis, HeLa cells were synchronized in G1/S phase by double thymidine block and then released into fresh media containing either MK-1775 or a solvent control (DMSO) (Figure 1A). Cells were fixed 4 h after treatment with MK-1775 and then examined for phospho-serine 10 histone H3 (PH3), which is a mitotic specific biomarker [25], by immunofluorescence microscopy (Figure 1B). Approximately 20% of cells were positive for PH3 following MK-1775 treatment compared to ∼1% in DMSO controls (Figure 1B and 1C, student *t*-test, *p <* 0.05). Cells that stained positive for PH3 also had condensed DNA as observed by DAPI staining consistent with a mitotic morphology. We also treated three different breast cancer cell lines (MDA-MB-231, T-47D, and MCF7) and one non-tumorigenic breast cell line (MCF 10A) with MK-1775 following G1/S synchronization (Figure 1C). The molecular subtype and p53 status for cell lines is indicated in Table 1. We observed that MK-1775 treatment increased the percentage of PH3-positive cells in HeLa (*p <* 0.005), T-47D (*p <* 0.005), and MDA-MB-231 (*p <* 0.05) to a similar level (∼20%) compared to DMSO controls; the percent of PH3-positive cells also increased for MCF7 cells (*p <* 0.05), but to a lesser extent (∼5%) (student *t*-test). No significant change in the percent of PH3 positive cells was observed in MCF 10A cells treated with MK-1775 compared to DMSO. We confirmed that siRNA knockdown of Wee1 in G_1_/S synchronized HeLa cells also increased PH3 staining compared to scrambled siRNA (siSc) controls (Figure 2; student *t*-test, *p <* 0.05). To confirm visual results, we also analyzed cells by flow cytometry. Cells were treated with MK-1775 or DMSO and then fixed and stained for PH3, and DNA after 4–8 h (Figure 1D and Supplementary Figure 1). We observed 25-29% of cells treated with MK-1775 were positive for PH3 during the 4-8 h treatment, whereas < 2% of cells treated with DMSO were positive for PH3 at any time (Figure 1D). Based on DNA content, we confirmed that two-thirds of the MK-1775 treated cells that were positive for PH3 staining had less than 4N DNA. Together, these data confirm that inhibition of Wee1 kinase induces premature mitosis from G1/S phase.

**Table 1:** p53 status and molecule subtypes of cell lines

Cell line	Cell type (ATCC)	Tissue type (ATCC)	p53 status	Molecular sub-type
**HeLa**	Epithelial	Cervical	Wild type HPV E6 deactivated	N.A.
**MDA-MB-231**	Epithelial	Mammary gland/breast	Missense R273H	Basal (triple negative)
**MDA-MB-468**	Epithelial	Mammary gland/breast	Missense R280K	Basal (triple negative)
MCF 10A*	Epithelial	Mammary gland/breast	Wild type	Basal (triple negative)
**MCF7**	Epithelial	Mammary gland/breast	Wild type	Luminal A ER+/PR+
**T-47D**	Epithelial	Mammary gland	Missense L194F	Luminal A ER+/PR+

p53 status for breast cancer cell lines is reviewed in [48]. Molecular subtypes were assigned based on ATCC classifications.

*represents non-tumourigenic cell line.

Knowing that inhibiting Wee1 induced premature entry into mitosis from G1/S phase, we tested if inhibiting other kinases involved in either the entry into or exit from mitosis would affect the number of PH3-positive cells observed by immunofluorescence. We released cells from G1/S into media containing UCN-01 (Chk1 inhibitor), AZ-3146 (Mps1 inhibitor), and CR8 (Cdk1 inhibitor) alone or in the presence of MK-1775 for 4 h (Supplementary Figure 2). Of the listed inhibitors used as a single agent, only MK-1775 treatment enhanced the number of PH3 positive cells (∼26%) compared to DMSO control (∼0.5%) (One-way ANOVA and Dunnett’s multiple comparisons test, *p <* 0.0001). Co-treatment with both UCN-01 and MK-1775 increased the percent of PH3-positive cells compared to MK-1775 treatment alone (∼33% verses ∼26%) (One-way ANOVA and Dunnett’s multiple comparisons test, *p <* 0.05). In contrast, CR8 treatment repressed the percent of PH3-positive cells when combined with MK-1775 compared to MK-1775 alone (∼5% vs ∼26%) (One-way ANOVA and Dunnett’s multiple comparisons test, *p <* 0.0001). AZ-3146 had no significant effect when combined with MK-1775 compared to MK-1775 alone. These results confirm that the Wee1 induced premature mitosis is dependent on Cdk1 activity and that an increased mitotic index can be stimulated by co-inhibiting Chk1, a kinase that is an upstream positive regulator of Wee1.

### Wee1 but not Myt1 kinase activity is required to prevent premature mitosis in HeLa cells

Since both Wee1 and Myt1 add inhibitory phosphates to Cdk1, we asked if the loss of both Wee1 and Myt1 kinase activity was required to induce premature mitosis. MK-1775 is reported to be 100 times more potent against Wee1 than Myt1, but at concentrations above 300 nM MK-1775 both kinases may be inhibited [15]. To determine if the loss of both kinase activities was required for premature mitosis, we knocked-down Wee1 and Myt1 alone or in combination by siRNA in cells blocked in G1/S phase (Figure 2). We found that 11% of cells transfected with siRNA against Wee1 expressed PH3 whereas only 4% of cells transfected with scrambled control siRNA (siSc) expressed PH3 (Figure 2C; student *t*-test, *p <* 0.05). In contrast to cells that had Wee1 knocked down, the number of cells positive for PH3 when Myt1 was knocked down did not differ from scramble controls. Furthermore, the combined knockdown of Wee1 and Myt1 kinases did not enhance PH3 staining compared to the knockdown of Wee1 alone (8% vs 11%). We confirmed the knockdown efficiencies of Wee1 and Myt1 protein levels by western blotting was approximately 80% for each protein (Supplementary Figure 3A–3C; student *t*-test, *p <* 0.0058 and *p =* 0.0009 for siMyt1 and siWee1 respectively). In addition, we also observed a decrease in the phosphorylation of tyrosine 15 and threonine 14 on Cdk1 following the knockdown of Wee1 and Myt1 respectively (Supplementary Figure 3D–3E, student *t*-test, *p <* 0.0005 and *p =* < 0.05). These data suggest that Wee1 has a more dominant role in regulating Cdk1 activity in HeLa cells compared to Myt1.

### Loss of Wee1 activity induces centromere fragmentation

**Figure 3:**
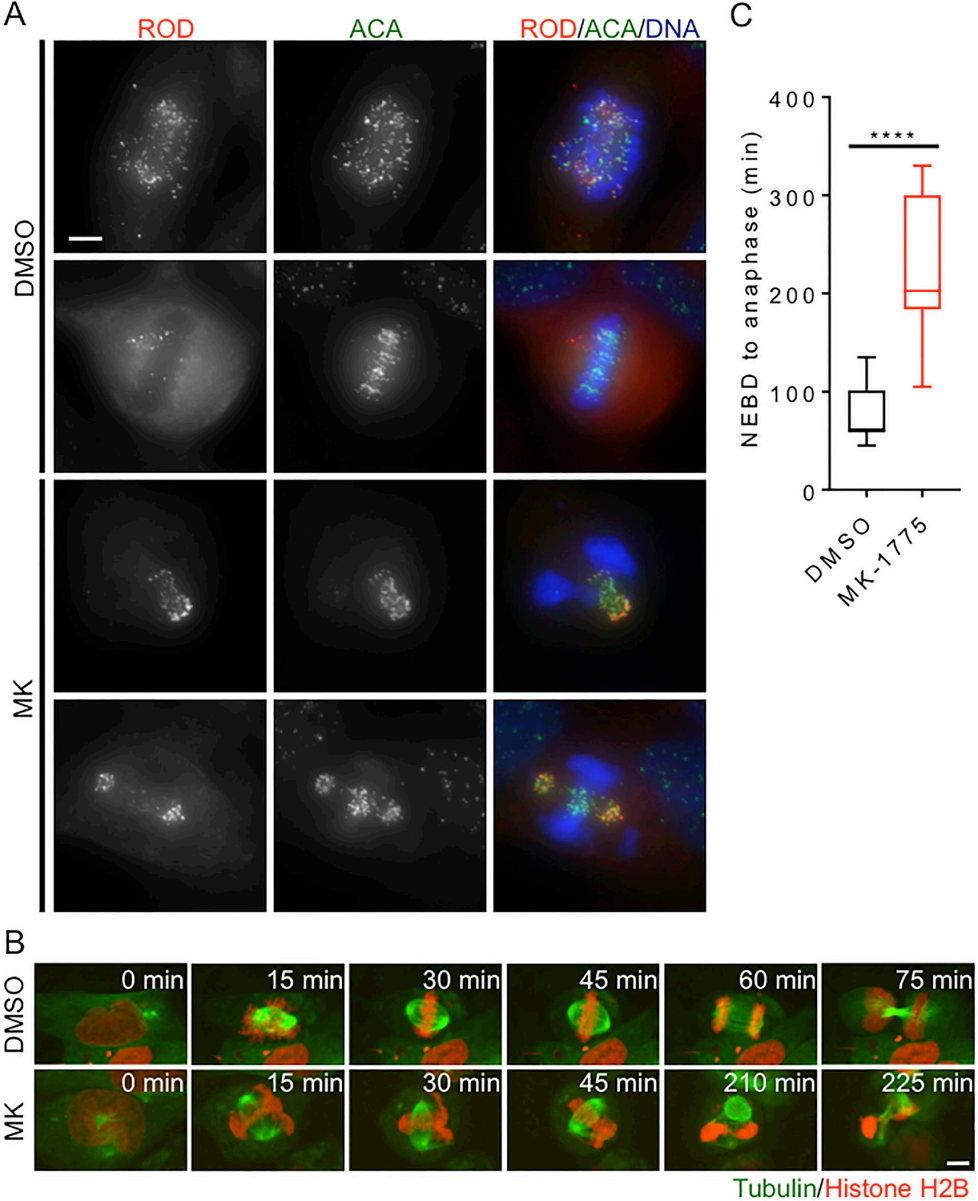
Inhibition of Wee1 induces centromere fragmentation and prolonged mitosis HeLa cells were synchronized in G1/S phase and then released into media containing either DMSO or MK-1775 (MK). (**A**) Mitotic cells fixed 4 h post treatment with MK were compared to mitotic cells fixed post treatment with DMSO. Cells were stained for Rough deal (ROD), anti-centromere antibody (ACA), and DNA. Scale bar = 10 μm. (**B**) HeLa cells stably expressing mCherry-H2B and EGFP-tubulin were treated with DMSO and MK and then analyzed by time-lapse microscopy to determine duration of mitosis (scale bar = 8 µm). Time is counted from NEBD in minutes. MK treated cell (lower panel) is also depicted as cell 12 in Supplementary Figure 4B. (**C**) The duration of mitosis (NEBD to anaphase) is shown for 8 cells for each treatment. Boxes represent interquartile distributions and whiskers represent 10th and 90th percentiles. Statistical significance was determined by student *t*-test (****p < 0.0001).

Having confirmed that loss of Wee1 activity induces premature entry into mitosis, we next examined the cellular morphology of MK-1775 treated cells that enter mitosis. Previous groups have reported that failure to complete DNA synthesis prior to mitotic entry can induce abnormal DNA condensation leading to torsional strain along the DNA backbone and centromere fragmentation [20, 21]. We asked if cells treated with MK-1775 also exhibited centromere fragmentation. We fixed cells released from G1/S phase 4 h after MK-1775 treatment and stained for the mitotic checkpoint protein Rough Deal (Rod) [26], centromeres (ACA), and DNA (Figure 3A). In both MK-1775 and DMSO treatment, we found that Rod staining overlapped with ACA staining confirming activation of the mitotic checkpoint [31]; however, the majority of the Rod/ACA in MK-1775 treated cells was clustered away from the main mass of chromosomes suggesting centromere fragmentation had occurred. We confirmed that siRNA knockdown of Wee1 also resulted in the centromere fragmentation morphology observed in MK-1775 treated cells (Supplementary Figure 3F), which supports that observed morphology was dependent on Wee1 and not another off-target kinase.

We then asked if MK-1775 induced centromere fragmentation affected the ability of cells to complete mitosis. We released HeLa cells (stably transfected with mCherry-H2B and EGFP-tubulin) from G1/S phase and then monitored the time cells spent in mitosis by time-lapse microscopy (Figure 3B). We found that cells released into MK-1775 remained in mitosis for a median time of 203 min whereas DMSO treated cells only stayed in mitosis for 60 min (Figure 3C, student *t*-test, *p <* 0.0001). Perhaps not surprisingly, most MK-1775 treated cells did not achieve chromosome alignment and exited mitosis without chromosome segregation (Supplementary Figure 4A). We tracked MK-1775 treated cells that underwent centromere fragmentation for up to 24 hours after they had exited mitosis and found that 86% (6/7) of these cells subsequently died in interphase (Supplementary Figure 4B, cells labelled 11–17). These data suggest that MK-1775 treatment prolongs mitosis by disrupting normal chromosome segregation, which ultimately leads to cell death.

### Inhibition of Wee1 prevents normal mitotic exit

**Figure 4:**
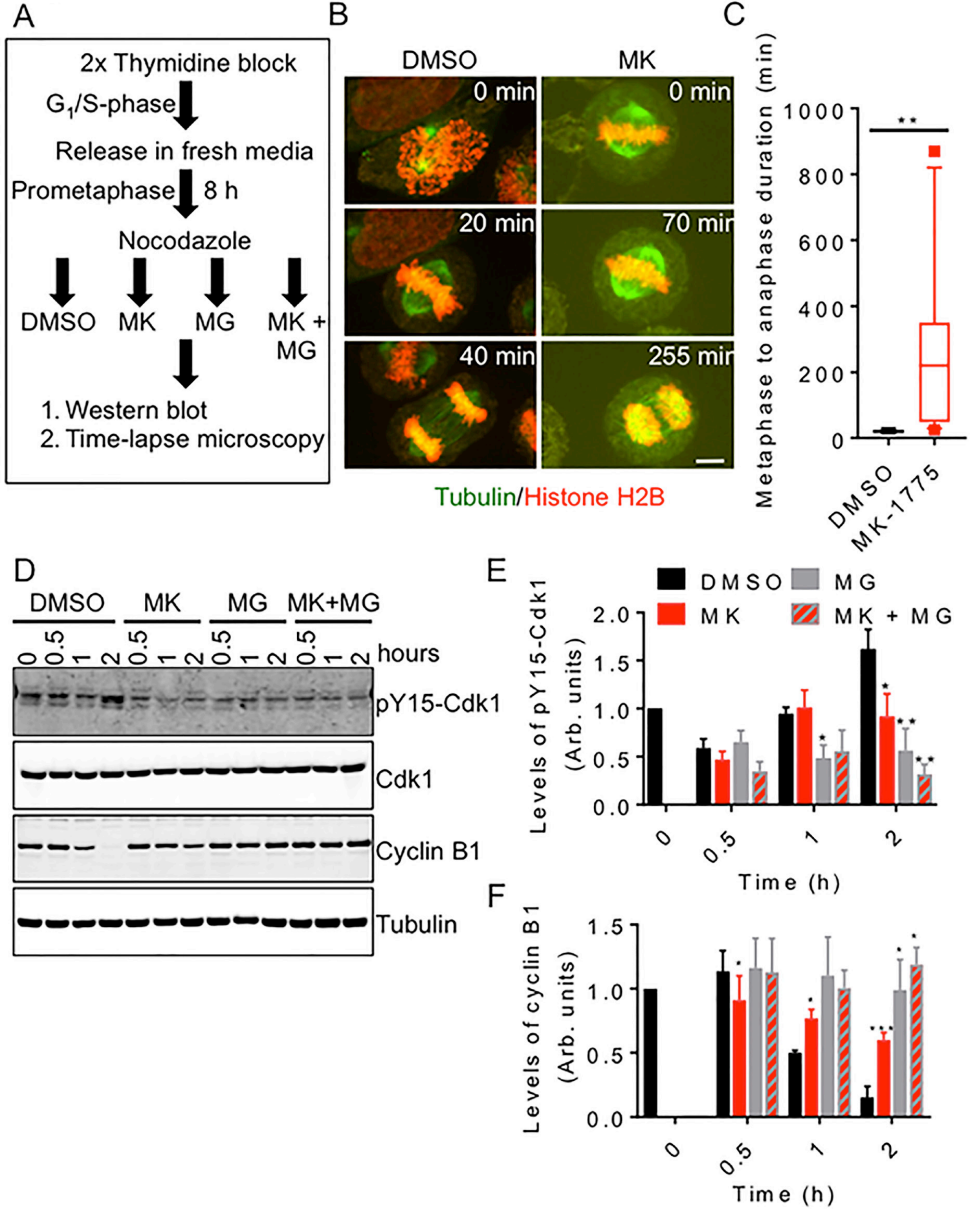
Inhibition of Wee1 prevents normal mitotic exit HeLa cells were synchronized and then released into media containing DMSO, MK-1775 (MK), MG-132 (MG) or both MK and MG for two hours. (**A**) Experimental flow chart of treatment conditions. (**B**) HeLa cells stably expressing mCherry-H2B and EGFP-tubulin were released into DMSO and MK and monitored by time-lapse microscopy. Scale bar = 8 µm. (**C**) The duration of the transition between metaphase and anaphase was measured for 12 cells for each treatment. Boxes represent interquartile distributions and whiskers represent 10th and 90th percentiles. Square dots represent outliers. Statistical significance was determined by student *t*-test (**p < 0.005). (**D**) Cell extracts were prepared from treated cells at indicated times. Extracts were then analyzed by western blot for the levels of pY15-Cdk1, Cdk1, cyclin B1, and tubulin. (**E**) Average levels of pY15-Cdk1 (relative to Cdk1) and (**F**) cyclin B1 (relative to tubulin) were measured over time. Levels of pY15-Cdk1 and Cyclin B1 at time zero were set as 1. Error bars represent standard error of the mean. Statistical significance was determined using One-way ANOVA and Dunnett’s multiple comparisons test (*p < 0.05, **p < 0.005 and *** *p <* 0.0005). Experiments were repeated at least three times.

Knowing that Wee1 activity is required for normal mitosis in cells released from G1/S phase, we then asked if Wee1 activity was also required for normal mitosis in cells released from prometaphase. We synchronized HeLa cells (stably transfected with mCherry-H2B and EGFP-tubulin) in prometaphase using nocodazole treatment (Figure 4A). We then released cells from nocodazole treatment into media containing either MK-1775 or DMSO and then measured the time required for cells to complete anaphase (Figure 4B and 4C). We found that the average time required to complete mitosis (metaphase to anaphase) was similar among DMSO treated cells (Figure 4C); however, we found that MK-1775 treated cells arrested at metaphase (Figure 4B, right set of images). During this metaphase arrest, many cells experienced one or more spindle collapses resulting in the temporary loss of chromosome alignment. Overall, we found that the transition from metaphase to anaphase in cells treated with MK-775 took a median time of 220 min with some cells requiring greater than 700 min to initiate anaphase (Figure 4C). In contrast, DMSO treated cells took only 20 min to transition from metaphase to anaphase (student *t*-test, *p =* 0.0023). To confirm the delay in mitotic exit, we also released cells synchronized in prometaphase into media containing either MK-1775 or DMSO for up to 2 h and then analyzed the levels of cyclin B1 and phospho-tyrosine 15 Cdk1 (pY15-Cdk1) by western blotting (Figure 4D–4F). We found that MK-1775 treated cells have reduced pY15-Cdk1 and retained 60% of cyclin B1 after 2 h compared to DMSO controls, which had only 15% of cyclin B after 2 h (One-way ANOVA and Dunnett’s multiple comparisons test, *p <* 0.0005). We also tested if inhibition of proteasome activity with MG-132 [32] could further enhance the effects of MK-1775. Although MG-132 treatment resulted in both higher cyclin B1 and lower pY15-Cdk1 levels compared to DMSO treatment, the differences were not found to be significant (One-way ANOVA and Dunnett’s multiple comparisons test, *p =* 0.1246). No significant differences were observed between MK-1775 versus the co-treatment with MG-132 (Figure 4E and 4F). We also confirmed that treatment with MK-1775 inhibited Cdk1 phosphorylation and cyclin B1 degradation in U-2 OS, T-47D, and MDA-MB-231 cell lines (Supplementary Figure 5). Together these data confirm that Wee1 activity is required for normal mitotic exit from prometaphase through a Cdk1/cyclin B1-dependent pathway.

### Prolonged mitosis induced by Wee1 inhibition enhances cell death

**Figure 5:**
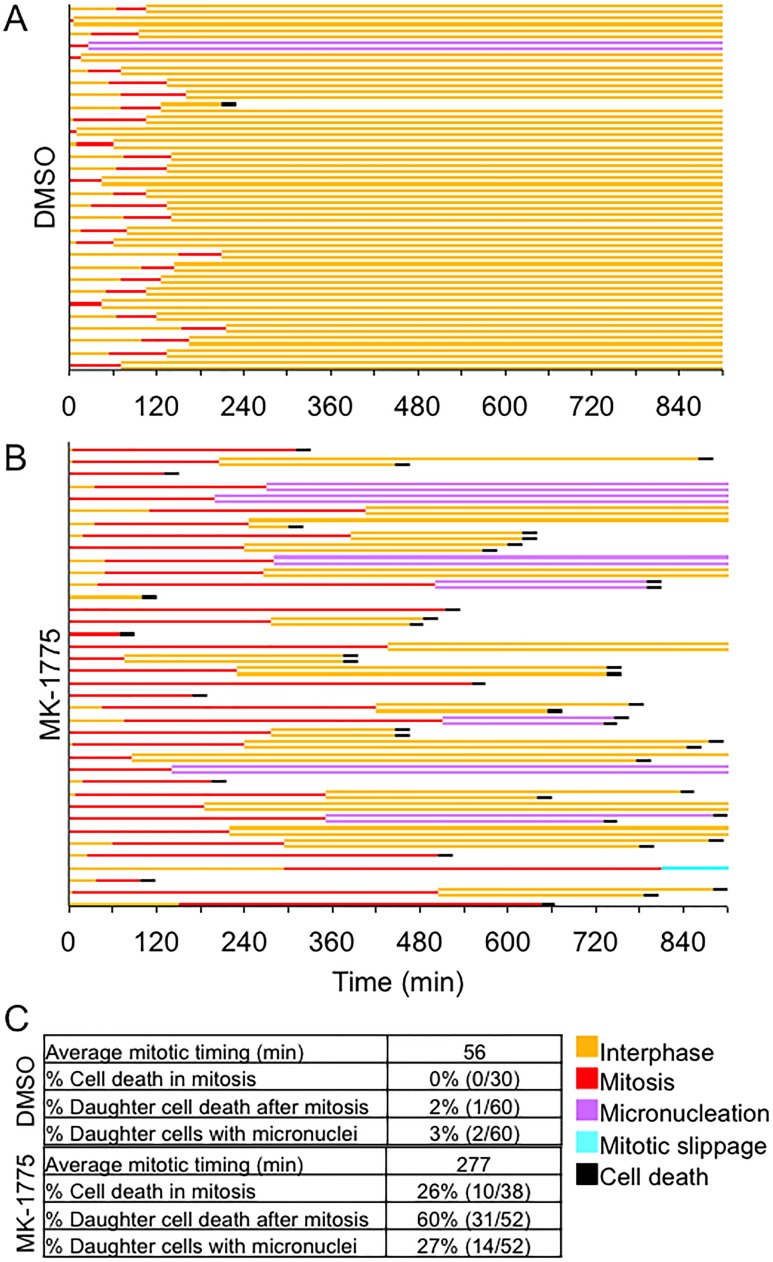
MK-1775 induced mitotic arrest promotes cell death HeLa cells expressing EGFP-H2B were released from double thymidine block for 9 hours and then treated with either (**A**) DMSO or (**B**) MK-1775 for 900 min. A line graph for 38 individual mitotic cells tracked by time-lapse microscopy is shown, which includes times for indicated cellular events. A fork in the line indicates cell division and cell fate of daughter cells is also shown. (**C**) Table shows the average time spent in mitosis (NEBD to anaphase) and the percentage of cells classified as having indicated features.

Since inhibition of Wee1 in prometaphase cells extended mitosis compared to controls, we then asked if increased mitotic time induced by MK-1775 led to an increase in cell death. We treated mitotic synchronized HeLa cells (expressing EGFP-H2B) with either MK-1775 or DMSO and then examined cell fate by time-lapse microscopy for up to 900 min (Figure 5). We found that 26% (10/38) of mitotic cells examined, died without completing anaphase. We observed successful completion of mitosis in 68% (26/38) of cells monitored, but 27% (14/52) of resulting daughter cells exhibited extra-nuclear structures or micronucleation and 60% (31/52) of the daughter cells died in interphase several hours after anaphase. In contrast, cell death or cells with micronuclei were rarely detected in DMSO controls. These data confirm that the prolonged mitosis induced by MK-1775 also induces cell death.

### Paclitaxel treatment enhances MK-1775 mediated cell killing

**Figure 6:**
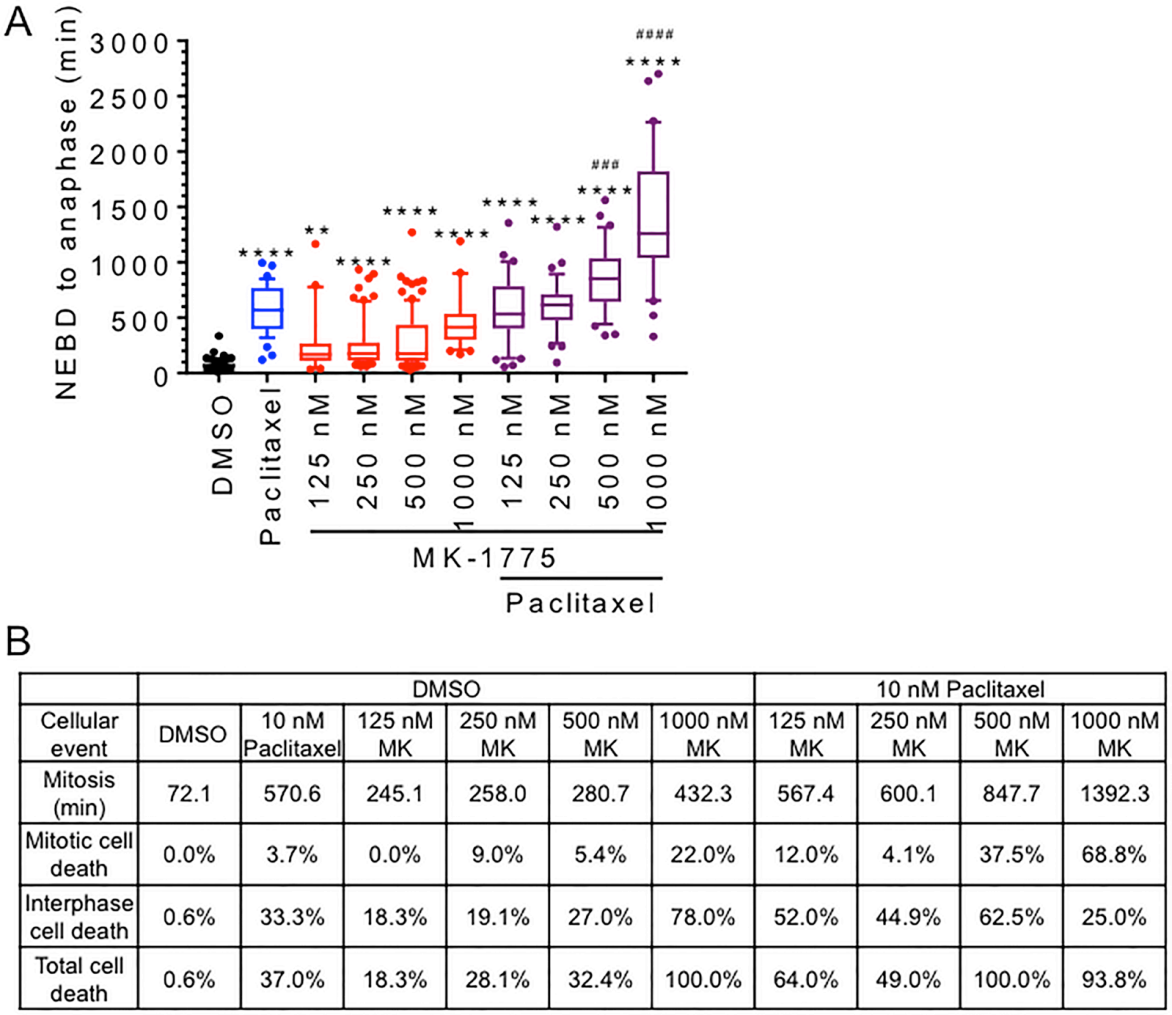
MK-1775 induced mitotic arrest is enhanced by co-treatment with paclitaxel HeLa cells stable expressing GFP-H2B were released from double thymidine block for 9 hours and then treated with DMSO, 10 nM paclitaxel, MK-1775 at indicated concentration alone, or both paclitaxel and MK-1775 for 48 h. (**A**) Duration of mitosis (NEBD to anaphase) is shown for at least 30 cells for indicated treatments. Boxes represent interquartile distributions and whiskers represent 10th and 90th percentiles. Coloured dots represent statistical outliers. Statistical significance was determined by One-way ANOVA and Tukey’s comparisons test. Asterisk (*) corresponds to significance between DMSO and indicated treatments (**p < 0.005 & ****p < 0.0001), whereas hashtag (^#^) corresponds to significant increases between co-treatments with paclitaxel and MK-1775 compared to paclitaxel alone (^###^*p <* 0.0005 & ^####^*p <* 0.0001). Cell fate dendrograms corresponding to DMSO, 500 nM MK-1775, 10 nM paclitaxel, or 500 nM MK-1775 and 10 nM Paclitaxel are included in Supplementary Figure 6. (**B**) Table shows the average duration of mitosis (NEBD to anaphase) and the percentage of cell death (mitosis or interphase) for indicated treatments.

Knowing that Wee1 is required for normal mitotic exit, we tested if co-treatment with the anti-microtubule drug paclitaxel could prolong mitosis in Wee1 inhibited cells. Mitotic synchronized HeLa cells were treated with 10 nM paclitaxel alone or in the presence of different concentrations of MK-1775 (125 nM to 1000 nM) (Figure 6 and Supplementary Figure 6). Mono-treatments of both paclitaxel and MK-1775 (at all tested concentrations) increased the average mitotic transit time (NEBD to anaphase) compared to DMSO controls. The average mitotic transit time in the presence of paclitaxel was found to be longer compared to mono-treatments of MK-1775 at concentration of 125 nM to 500 nM (One-way ANOVA and Tukey’s multiple comparisons test, *p <* 0.0001), but no difference was observed between paclitaxel and 1000 nM MK-1775. We also observed abnormal spindle formation (tri-polar spindle) with paclitaxel treatment alone consistent with previous groups [28]. We found that combined treatments with paclitaxel and MK-1775 (500 nM to 1000 nM) prolonged average mitotic transit time relative to either treatment alone (One-way ANOVA and Tukey’s multiple comparisons test, *p <* 0.0005 and *p <* 0.0001). In addition, we observed that co-treatments increased the percentage of cell death that occurred in both mitosis and the subsequent interphase (Figure 6B). Mono-treatments of paclitaxel and 500 nM MK-1775 resulted in 37% and 32% cell death respectively, however, combination treatments with both paclitaxel and 500 nM MK-1775 resulted in the death of all cells monitored (Figure 6B and Supplementary Figure 6). Of the total cell death observed, we found that co-treatments with both MK-1775 and paclitaxel resulted in more cell death occurring in mitosis. We observed that mono-treatments with 1000 nM MK-1775 resulted in 100% cell death (Figure 6B), but the addition of paclitaxel increased the percent of cell death in mitosis from 22% to 68.8% and reduced the percent of cell death in interphase from 78% to 25%. This data support that prolonging the Wee1 induced mitotic arrest can be used to enhance cell killing.

**Figure 7:**
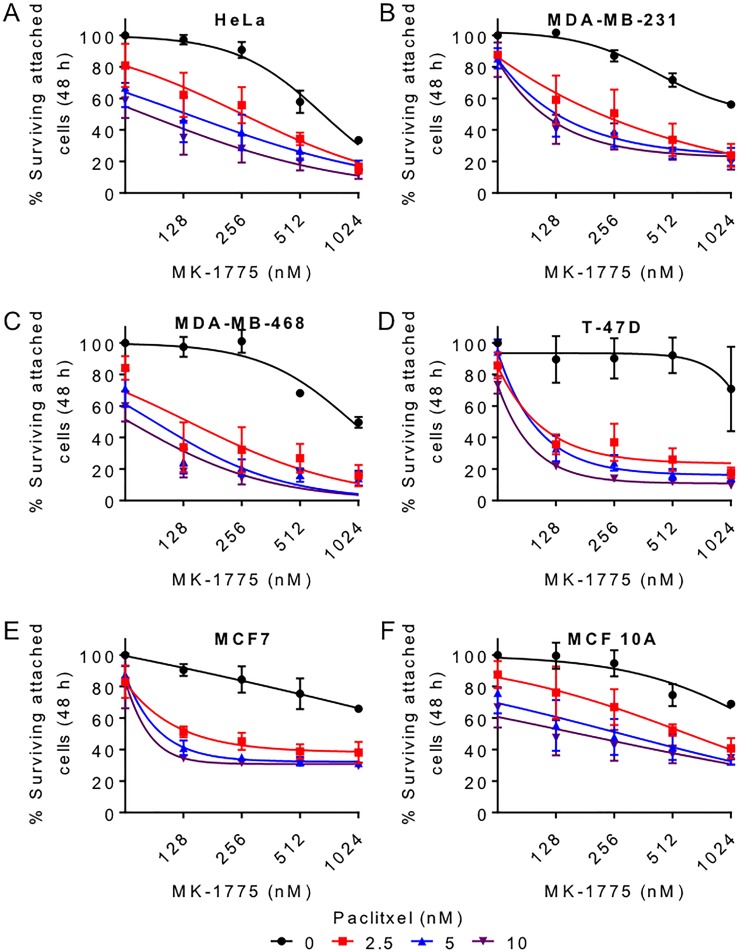
Paclitaxel enhances MK-1775 mediated cell killing in breast cancer cells (**A**) HeLa, (**B**) MDA-MB-231, (**C**) MDA-MB-468, (**D**) T-47D, (**E**) MCF7, and (**F**) MCF 10A cells were released from G1/S phase for 9 h and then treated with increasing concentration of MK-1775 alone (black curve) or in the presence of 2.5 nM (red curve), 5 nM (blue curve), or 10 nM (purple curve) paclitaxel for 48 h. Average percent surviving attached cells (% Crystal violet OD) is shown. The first point on each curve represents 0 nM MK-1775. Error bars represent standard error of mean (SEM). Statistical significance was determined by Two-way ANOVA (See Table 2).

Since combination treatments with MK-1775 and paclitaxel led to a greater mitotic transit time and increased cell death in HeLa cells, we then tested if combining paclitaxel and MK-1775 could enhance cell killing in different breast cancer cell lines (T-47D, MDA-MB-231, MDA-MB-468, and MCF7). We first treated the different cell lines with different concentrations of MK-1775 (125–1000 nM) alone or in the presence of different concentrations of paclitaxel (2.5–10 nM) for 48 h. We then measured the average percent survival (percent crystal violet OD) of attached cells by crystal violet assay (Figure 7). Alone, both paclitaxel and MK-1775 significantly reduced the percentage of surviving attached cells as determined by a Two-way ANOVA test (Table 2, see the first two *p* values corresponding to MK-1775 and paclitaxel for the indicated cell lines). As predicted the combination of paclitaxel and MK-1775 also had a significant interaction on cell survival in HeLa and the four breast cancer cell lines tested (Figure 7A–7E and Table 2, see third *p* value (interaction) for the corresponding cell lines). We also tested co-treatments with MK-1775 and paclitaxel in a non-tumorigenic breast cell line (MCF 10A), but we did not observe a significant interaction between MK-1775 and paclitaxel (Figure 7F and Table 2). These data support that inhibition of Wee1 in the presence of paclitaxel can enhance cell killing in breast cancer cell lines.

**Table 2:** Paclitaxel and MK-1775 co-treatment interaction in cells significantly reduces cell survival

**HeLa**					
ANOVA table	SS	DF	MS	F (DFn, DFd)	*P* value
MK-1775	31590	4	7898	F (4, 12) = 31.43	*P* < 0.0001
Paclitaxel	23037	3	7679	F (3, 9) = 15.63	*P* = 0.0007
Interaction: MK-1775 × Paclitaxel	3120	12	260	F (12, 36) = 7.372	*P* < 0.0001
Interaction: MK-1775 × Subjects	3016	12	251.3		
Interaction: Paclitaxel × Subjects	4423	9	491.4		
Subjects	9603	3	3201		
Residual	1270	36	35.27		
**MDA-MB-231**					
ANOVA table	SS	DF	MS	F (DFn, DFd)	*P* value
MK-1775	32916	4	8229	F (4, 12) = 29.33	***P* < 0.0001**
Paclitaxel	22250	3	7417	F (3, 9) = 16.53	***P* = 0.0005**
Interaction: MK-1775 × Paclitaxel	3039	12	253.3	F (12, 36) = 4.076	***P* = 0.0005**
Interaction: MK-1775 × Subjects	3367	12	280.6		
Interaction: Paclitaxel × Subjects	4039	9	448.7		
Subjects	6514	3	2171		
Residual	2237	36	62.14		
**MDA-MB-468**					
ANOVA table	SS	DF	MS	F (DFn, DFd)	*P* value
MK-1775	21388	4	5347	F (4, 8) = 40.47	***P* < 0.0001**
Paclitaxel	32402	3	10801	F (3, 6) = 24.76	***P* = 0.0009**
Interaction: MK-1775 × Paclitaxel	4878	12	406.5	F (12, 24) = 4.922	***P* = 0.0004**
Interaction: MK-1775 × Subjects	1057	8	132.1		
Interaction: Paclitaxel × Subjects	2618	6	436.3		
Subjects	1342	2	671.1		
Residual	1982	24	82.58		
**T-47D**					
ANOVA table	SS	DF	MS	F (DFn, DFd)	*P* value
MK-1775	26498	4	6624	F (4, 8) = 23.76	***P* = 0.0002**
Paclitaxel	34684	3	11561	F (3, 6) = 23.64	***P* = 0.0010**
Interaction: MK-1775 × Paclitaxel	5181	12	431.7	F (12, 24) = 3.288	***P* = 0.0063**
Interaction: MK-1775 × Subjects	2230	8	278.8		
Interaction: Paclitaxel × Subjects	2935	6	489.1		
Subjects	2235	2	1118		
Residual	3152	24	131.3		
**MCF7**					
ANOVA table	SS	DF	MS	F (DFn, DFd)	*P* value
MK-1775	11633	4	2908	F (4, 4) = 30.78	***P* = 0.0029**
Paclitaxel	10673	3	3558	F (3, 3) = 15.42	***P* = 0.0251**
Interaction: MK-1775 × Paclitaxel	1330	12	110.8	F (12, 12) = 4.56	***P* = 0.0068**
Interaction: MK-1775 × Subjects	378	4	94.5		
Interaction: Paclitaxel × Subjects	692.3	3	230.8		
Subjects	195	1	195		
Residual	291.6	12	24.3		
**MCF 10A**					
ANOVA table	SS	DF	MS	F (DFn, DFd)	*P* value
MK-1775	10581	4	2645	F (4, 8) = 30.49	***P* < 0.0001**
Paclitaxel	15637	3	5212	F (3, 6) = 8.241	***P* = 0.0150**
Interaction: MK-1775 × Paclitaxel	1026	12	85.5	F (12, 24) = 1.408	*P* = 0.2291
Interaction: MK-1775 × Subjects	694.1	8	86.76		
Interaction: Paclitaxel × Subjects	3795	6	632.5		
Subjects	4908	2	2454		
Residual	1458	24	60.73		

Cells were treated with increasing concentration of MK-1775 alone or in the presence of increasing concentrations of paclitaxel (see Figure 7). Table presented for each cell line shows statistical analysis of treatments determined by Two-way ANOVA. SS, DF, MS, FDn, and FDd are defined as sum of squares, degrees of freedom, mean square, degrees of freedom numerator, and degrees of freedom dominator respectively.

## DISCUSSION

### MK-1775 induces centromere fragmentation, a key morphological feature of mitotic catastrophe

Wee1 kinase is a pivotal negative regulator of Cdk1/cyclin B1 activity and is required for normal entry into and exit from mitosis. We find that loss of Wee1 activity promotes both premature mitosis and a prolonged mitotic arrest leading to cell death. Normally, HeLa cells that are released from G1/S phase require 10–12 h to complete DNA replication and to synthesize other crucial biomolecules before entering mitosis [29]. However, in our experiments after 4 h of treatment with MK-1775, 25% of G1/S-synchronized HeLa cells entered mitosis and two-thirds of these cells had < 4N DNA. MK-1775 also induced premature mitosis in MDA-MB-231 and T-47D cells similarly to HeLa cells (all ∼20%). We observed premature mitosis in 5% of MCF7 cells but no changes in MCF 10A cells treated with MK-1775. The reduced (or lack of) premature mitosis observed in MCF7 and MCF 10A cells compared to the other cancer cell lines tested following Wee1 inhibition may be because both MCF7 and MCF 10A cell lines have wild-type p53, which has been shown to reduce cell sensitivity to MK-1775 [15, 19]. This finding confirms that these cells entered mitosis prematurely without completing DNA replication. Premature mitosis is typically associated with mitotic catastrophe, a mode of cell death that is yet to be defined by a molecular pathway [22]. In addition to premature mitosis, mitotic catastrophe is also associated with centromere fragmentation, micronucleation, and prolonged mitosis [21, 22].

We characterized the morphology of the premature mitotic cells and found that they exhibited key features of centromere fragmentation [21]. Centromere fragmentation has been reported to occur in both Chinese hamster ovarian [20] and human cell lines [21] that are forced into mitosis with damaged DNA from an S-phase arrest. Cells treated with MK-1775 that underwent premature mitosis had centromeres (marked by ACA) and kinetochore proteins (marked by Rod) that co-clustered away from condensed DNA (Figure 3A). This finding is consistent with that reported by Beeharry *et al.*, who reported that during centromere fragmentation, the centromeres and kinetochore proteins such as Mis12, Aurora B, and CENP-F co-clustered away from the bulk of the condensed DNA in pancreatic cancer cells [21]. Furthermore, our study and a previous study showed that cells that survive treatment with MK-1775 often exhibit micronuclei or micronucleation [19], which is a consistent outcome for cells that have previously undergone an abnormal mitosis [30], such as centromere fragmentation. Our data confirms for the first time that loss of Wee1 activity in G1/S synchronized cells induces centromere fragmentation, which prevents normal chromosome alignment and segregation.

### Wee1 and Myt1 are not functionally redundant in HeLa cells

We tested if Myt1, like Wee1 induces premature mitosis or centromere fragmentation. Myt1 is shown to have functional redundancy with Wee1 in *Drosophila* and is known to regulate Cdk1 through inhibitory phosphorylation on threonine 14 and tyrosine 15 [31]. However, we did not detect any change in the mitotic index when Myt1 levels were reduced to ∼12% by siRNA transfection alone or when knocked-down with Wee1 (Figure 2 and Supplementary Figure 3). Our results are consistent with others who have reported that Myt1 knockdown does not affect either entry or exit of mitosis in HeLa cells [32, 33], which suggests that the lack of Myt1 activity likely does not play a role in centromere fragmentation.

### MK-1775 induces cell death in a way similar to anti-mitotic drugs

We confirmed that Wee1 inhibition prolongs mitosis [6–8, 10, 34], and we show that increased time in mitosis results in cell death. We found that cells released from both G1/S phase and prometaphase exhibited longer times in mitosis (Figure 3B and 3C), which is commonly observed during mitotic catastrophe. Cells that exhibited centromere fragmentation remained in mitosis for several hours before exiting and subsequently dying (Supplementary Figure 4). However, while in mitosis Rod was localized to the kinetochore (Figure 3A) suggesting mitotic checkpoint activation [26, 35]. Prolonged activation of the mitotic checkpoint and delayed mitotic exit has been shown to occur when there are chromosome attachment errors [36]. Therefore, part of the prolonged mitosis observed in cells exhibiting centromere fragmentation was likely because normal bipolar chromosome attachment could not be achieved. However, part of the prolonged mitosis can also be attributed to the loss of Wee1 activity [6–8, 10, 34]. In support of this, we found that prometaphase synchronized cells with fully replicated chromosomes that were capable of normal bipolar attachment, still arrested in mitosis when treated with MK-1775. We used time-lapse microscopy and resolved that this arrest occurred in metaphase but was not due to centromere fragmentation. During the metaphase arrest cells experienced multiple spindle collapses, and overall were found to require ∼5 times longer to complete mitosis (or ∼12 times longer to transition from metaphase to anaphase) compared to DMSO controls (Figure 5). Importantly, the metaphase arrest induced by MK-1775 also enhanced cell death. We observed that 26% of cells died in mitosis and 60% of the progeny from cells that did complete mitosis died in interphase within ∼12 h of anaphase (Figure 5C). Cell death in both mitosis and interphase following prolonged mitosis has been previously documented in HeLa, A549, and HCT-116 cells that were treated with other anti-mitotic drugs such as nocodazole, paclitaxel, and a small molecule inhibitor of Eg5 (AZ-138) [23]. The driving mechanism of MK-1775 mediated cell killing has been suggested to be premature entry into mitosis from S-phase [15, 19]. However, our data show that MK-1775 also causes cell death by prolonging mitosis in cells that enter mitosis normally, which seems to be the cell death mechanism of other anti-mitotic drugs [23, 36, 37]. We also detected further evidence of mitotic catastrophe; 27% of daughter cells exhibited either micronucleation or contained extra-nuclear structures [22, 38], which likely arose because of repeated spindle collapses [39].

### Sustained Cdk1 activity prolongs mitosis leading to cell death

We believe that the inability to inhibit the Cdk1/cyclin B1 complex following chromosome alignment in cells treated with MK-1775 led to the observed metaphase arrest and subsequent cell death. We confirmed that cyclin B1 levels remained high and Cdk1 remained un-phosphorylated at tyrosine 15 in mitotic HeLa cells treated with MK-1775 (Figure 4D–4F). We also confirmed that there was stable cyclin B1 and low phospho-tyrosine 15 Cdk1 levels in other cell lines including U-2 OS, T-47D and MDA-MB-231 cells (Supplementary Figure 5). Our findings are consistent with those reported by Visconti *et al.* who also found high cyclin B1 and low phospho-tyrosine 15 Cdk1 levels 10 hours after cells were released from prometaphase into MK-1775. Additionally, HeLa cells transfected with a Wee1 phospho-mimetic inactive mutant (T239D), also fail to degrade cyclin B1 and phosphorylate tyrosine 15 of Cdk1 [7]. Together, our data show that MK-1775 can stimulate cell death by two separate means: inducing premature mitosis associated with centromere fragmentation and preventing mitotic exit through sustained Cdk1 activity.

### MK-1775 sensitizes breast cancer cells to paclitaxel

Since inhibition of Wee1 activity with MK-1775 prevents normal mitotic exit, we tested if co-treating cells with the microtubule poison paclitaxel could be used to enhance cell killing compared to treating cells with either paclitaxel or MK-1775 alone. Paclitaxel is used in adjuvant therapy in breast cancers as well as a single agent therapy for metastatic cancers. Importantly, paclitaxel treatment also prolongs mitosis, which has been suggested to be a driving mechanism of cell death [36, 37]. However, not all tumours respond to MK-1775 treatments, a fact that is also true for paclitaxel [40].

In our experiments, we examined the response of breast cancer cell lines to paclitaxel and MK-1775. We found that combining paclitaxel with MK-1775 (500–1000 nM) significantly increased total time in mitosis compared to either treatment alone in HeLa cells (Figure 6). We also observed an increase in the percentage of cells that died in mitosis in co-treated conditions compared to mono-treatments (Figure 6B). These experiments were consistent with crystal violet assay results, where we found that paclitaxel enhanced MK-1775 in four different breast cancer cell lines tested (T-47D, MDA-MB-231, MDA-MB-468, and MCF7) and vice versa (Figure 7B–7E) but not the non-tumourigenic breast cell line (MCF 10A). This might be because MCF 10A cells were less prone to undergoing premature mitosis compared to HeLa, MDA-MB-231, T-47D, and MCF7 cells when treated with MK-1775 (Figure 1D). Since MK-1775 did not enhance MCF 10A cell sensitivity to paclitaxel at the concentrations tested, combined treatments maybe more selective towards cancer cells, but this would need to be confirmed in additional normal cell lines. In either case, our findings are consistent with a recent study that showed MK-1775 sensitized human cell lines (HeLa, MCF7, and MOLT-4) and patient derived Acute Lymphoblastic Leukemia (ALL) cells to paclitaxel and other microtubule poisons (vincristine, and nocodazole) as marked by an increase in apoptosis and a reduction in cell viability [7]. Interestingly, breast cancer cells treated with paclitaxel are reported to down-regulate Wee1 expression [41], suggesting that loss of Wee1 activity is an important step in paclitaxel mediated-cell killing. Therefore, if paclitaxel induced cell death requires inactivation of Wee1, then it makes sense that co-treating cells with MK-1775 will result in additional cell killing.

Our data highlight a new potential strategy for enhancing MK-1775 mediated cell killing in breast cancer cells. Currently, paclitaxel is an approved treatment for breast cancer [42] and though MK-1775 is currently undergoing phase I/II clinical evaluations with different anti-cancer agents for the treatment of solid tumours affecting organs such as the cervix, ovaries, lungs, and pancreas, there are no dedicated studies examining the response of breast cancer to MK-1775 and paclitaxel (https://clinicaltrials.gov). Therefore, our data provide a rationale for combining MK-1775 and paclitaxel to treat breast cancer.

## MATERIALS AND METHODS

### Cell culture and synchronization

HeLa, U-2 O S, MDA-MB-468, and MDA-MD-231 cells were grown as a monolayer in high-glucose DMEM supplemented with 2 mM L-glutamine and 10% (vol/vol) FBS. T-47D cells were grown in RPMI1640 supplemented with 2 mM L-glutamine, 10% (vol/vol) FBS, 0.01 mg/ml insulin, and 1 mM sodium pyruvate. MCF7 cells were grown in high-glucose DMEM supplemented with 2 mM L-glutamine, 10% (vol/vol) FBS, and 0.01 mg/ml insulin. MCF 10A cells were grown in MEBM supplemented with SingleQuots (Lonza; CC-3150) (0.5 mL of gentamicin sulfate amphotericin B, 2 mL of bovine pituitary extract, 0.5 mL hydrocortisone, 0.5 mL epidermal growth factor (rHEGF), and 0.5 mL insulin). All cell lines were cultured in a humidified incubator at 37°C with 5% CO2.

### Small molecule inhibitors

All small molecule inhibitors were stored as 10 mM stock solutions in DMSO at -20^◦^C. Where applicable, cells were treated with 1 µM (unless otherwise indicated) MK-1775 (Chemie Tek; 955365-80-7), 1 mM UNC-01 (Sigma-Aldrich; 112953-11-4), 10 mM CR8 (Sigma-Aldrich; C3249-5MG), 2 µM AZ3146 (Selleckchem; s2731), 2.5-10 nM paclitaxel (Sigma; T7191).

### Cell synchronization

Cells were synchronized in G1/S phase by double thymidine block as previously described (Moudgil *et al.*, 2015) [43]. Cells were treated with 2 mM thymidine for 16 h with an 8 h release interval between thymidine treatments. Cell synchronization in prometaphase was performed 8 h post release from thymidine treatment by the addition of 200 ng/mL of nocodazole (Cell Signalling; 2190S) for 4 h. Cell synchronization described in Figures 5–7 (measuring NEBD to anaphase), cells were released from 2nd thymidine block for 9 h and then subjected to indicated treatments. Cell synchronization in metaphase was achieved using 25 µM MG-132 (Sigma-Aldrich; M7449).

### RNAi

siRNA for Wee1 (5′-CAUCUCGACUUAUUGGAAAtt-3′), Myt1 (5′-GGACAGCAGCGGAUGUGUUtt-3′) ora scrambled control siRNA (5′-UGGUUUACAUGUCGACUAA-3′) from Thermo Fisher Scientific were used at a concentration of 20 nM with 0.2% Lipofectamine RNAiMax (Thermo Fisher Scientific) for 24 h. siRNA transfections were initiated after the first thymidine block. Knockdown efficiency was analyzed by western blotting and normalized to tubulin levels.

### Western blotting

Cells were harvested and processed for western blot as described previously (Vos *et al.*, 2011) [44]. 10 µg of protein extract were separated on 12% polyacrylamide gels for 1 h at 150 V. PageRuler Plus Prestained protein ladder (Fermentas; Thermo Fisher Scientific) was used as a molecular weight marker. Proteins were transferred on to Polyvinylidene difluoride (PVDF) membrane (Bio-Rad Laboratories electroblotter system) for 17 h at 30 V. Membranes were blocked with Odyssey blocking buffer (LI-COR Biosciences). Membranes were probed with the following primary antibodies: anti-Wee1 antibodies (Santa Cruz; sc-5285; 1:200 dilution), anti-Myt1 antibodies (Cell Signalling; 4282; 1:300 dilution), anti-Cdk1 antibodies (Santa Cruz; sc-54; 1:500 dilution), anti-phospho-tyrosine 15 Cdk (Signalway Antibodies; 11244-2; 1:500 dilution), anti-phospho-threonine 14 Cdk (Cell Signalling; 2543; 1:1000 dilution), anti-tubulin antibodies (Sigma; T5168; 1:4000 dilution), and anti-cyclin B1 antibodies (Santa Cruz; sc-752; 1:200 dilution). Membranes were then incubated with Alexa Fluor—680 conjugated anti-rabbit (Thermo Fisher Scientific; A21109; 1:1000 dilution) or anti-mouse (Thermo Fisher Scientific; A21057; 1:1000 dilution). Membranes were scanned by Odyssey IR imager system (LI-COR Biosciences) and then analyzed by Odyssey V3.0 for quantification [45].

### Fluorescence microscopy

Cells were processed for immunofluorescence as previously described (Famulski *et al.*, 2011) [46]. Cells were seeded on to coverslips at a density of 5 × 10^4^ cells/ml in a 35-mm dish. Following cell synchronization cells were treated with the following inhibitors alone or in combination: 1 µM (unless otherwise indicated) MK-1775 (Chemie Tek; 955365-80-7), 1 μM UNC-01 (Sigma-Aldrich; 112953-11-4), 10 μM CR8 (Sigma-Aldrich; C3249-5MG), and 2 µM AZ3146 (Selleckchem; s2731). Treatments were maintained for 4 h and 0.1% DMSO was used as a control in all experiments. siRNA transfections were performed as outlined in the RNAi section. DNA was stained with 0.1 µg/ml DAPI. Coverslips were stained with the following antibodies: anti-phospho-Ser10 Histone H3 (PH3) antibodies (Abcam; ab5176; 1:1000 dilution), anti-tubulin antibodies (Sigma; T5168; 1:4000 dilution), Anti-centromere antibody (ACA) sera (gift from M. Fritzler, University of Calgary, Calgary, Canada; 1:4000 dilution), anti-Rod antibodies (N-terminal 809-aa antigen; Chan *et al.*, 2000; 1:1500 dilution). Alexa Fluor 488–conjugated anti–rat (1:1000 dilution; Molecular Probes), Alexa Fluor 555–conjugated anti–rabbit (1:1000 dilution; Molecular Probes), Alexa Fluor 555–conjugated anti–mouse (1:1000 dilution; Molecular Probes), and Alexa Fluor 647–conjugated anti–human (1:1000 dilution; Molecular Probes) secondary antibodies were used to visualize protein localization. Coverslips were mounted with 1 mg/ml Mowiol 4–88 (EMD Millipore) in phosphate buffer, pH 7.4. A microscope (Imager.Z.1; Carl Zeiss) equipped with epifluorescence optics was used to collect the images. Cells were visualized with a 100× Plan-Apochromat objective (Carl Zeiss) with 1.4 NA. Images were captured with a SensiCam QE charge-coupled device camera (PCO TECH, Inc.) controlled by Metamorph 7.0 software (Universal Imaging Corp.). Images were processed using Photoshop CC (Adobe).

### High-content imaging of mitotic index

Wide field fluorescence images were taken with a High-content automated microscopy imaging system (MetaXpress Micro XLS, software version 6, Molecular Devices, Sunnyvale, CA, USA). Briefly, at least 36 images per treatment (covering an area of ∼2 mm^2^/image) were taken with a 10× (NA 0.3) objective with the equipped siCMOS camera using bandpass filters of 447/60 nm for DAPI and 536/40nm for Alexa488 respectively. The images were then analyzed with the MetaXpress Cell scoring module which segments each cell nucleus using the DAPI signal. Mitotic cells were detected by PH3 staining. Positive PH3 stained cells were scored using a combination of criteria: 1) a minimal staining intensity threshold; 2) using DAPI as a mask for area staining; and 3) counted as percentage of the total number of nuclei (cells). On average, each slide yielded at least 20000-60000 cells.

### Live cell imaging

For analysis of mitotic timing, a HeLa cell line stably expressing EGFP-tubulin and mCherry H2B was used [43]. Cells seeded in a 35-mm glass-bottom dish (MatTek Corporation or Flourodish World Precision Instruments) were placed onto a sample stage within an incubator chamber maintained at a temperature of 37°C in an atmosphere of 5% CO_2_. Cell media was replaced with imaging media (OPTI-MEM; Gibco) supplemented with 2 mM L-glutamine, 10% (vol/vol) FBS, and 14 mM Hepes before imaging. Following releases from thymidine or nocodazole (see cell synchronization), cells were treated with either 1 µM MK-1775 or DMSO for up to 30 h.

Imaging was performed using a spinning disk (Ultraview, Perkin-Elmer) on an inverted microscope (Axiovert 200M; Carl Zeiss; with a 40× objective lens and 1.3 NA) equipped with an electron-multiplying charge-coupled device (EM-CCD) camera (ORCA-FLASH-4.0; Hamamatsu Photonics). Images were collected every 5 min for GFP and Cy3 channels using 100-ms exposure times, for 10–16 h using the Volocity software (Perkin-Elmer). Volocity 6.3.0 software was used to collect and export videos as AVI format using Microsoft video 1 compression. Videos were further converted to the mov format with Vegas Pro version 12.0 (Build 394; Sony Creative Software Inc.) using Sorenson 3 compression. Mitotic timing for cells was calculated manually. Still tiff format images from videos were exported using Volocity 6.3.0 software and processed using Photoshop CC. Statistical analysis was performed using GraphPad Prism version 5.04.

### Crystal violet assay

G1/S released cells were seeded into 96-well plates for 9 h and then treated with increases concentrations of MK-1775 (125–1000 nM, 1:2 series dilution) alone or in combination with paclitaxel (2.5, 5, or 10 nM) for 48 h. After treatment, media was aspirated and then cells were stained with 0.5% crystal violet assay for 20 min as outlined by Feoktistova *et al.* [47]. Crystal violet was later removed and plates were rinsed three times with water and left to air-dry for 24 h. Crystal violet stain was then resuspended in 100% methanol. Absorbance 570 nm (OD_570_) was measured using Optima Plate reader powered by Optima software. Percent surviving attached cells was calculated by subtracting off blank wells and then normalizing DMSO controls to 100%. The first point on each curve represents 0 nM MK-1775. Graphs were plotted using Graph Prisms V7.

### Flow cytometry

At desired times after treatment, cells treated with either 1 µM MK-1775 or DMSO were collected by aspiration and trypsinization. Cells were washed in PBS and fixed in 90% ethanol (-20^◦^C) for at least 24 h. Fixed cell suspensions were blocked for 1 h with labelling buffer (PBS, 5% serum, 1% BSA and 0.1% sodium azide) before 1 h of incubation with anti-phospho-Ser10- histone H3 (PH3) antibody (Abcam; ab5176; 1:1000 dilution), and 30 min of incubation with Alexa Fluor 488–conjugated anti–rabbit (Thermo Fisher Scientific; A-11034; 1:100 dilution) in labelling buffer, separated by wash/centrifuge steps in wash buffer (PBS, 1% BSA and 0.1% sodium azide). For analysis, samples were incubated for 20 min in wash buffer with 0.02 mg/ml propidium iodide (Invitrogen) and 0.2 mg/ml RNase A (Sigma), and analysed by a FACSCantoTM II flow cytometer (BD Biosciences) using BD FACSDivaTM software. Gating was set using control samples without primary antibody. Experiments were carried out three times.

## CONCLUSIONS

MK-1775 is a potent small molecule inhibitor that has two modes of cell killing. First, it can induce premature mitosis leading to centromere fragmentation in G1/S cells. Second, it induces a metaphase arrest because of dysregulated Cdk1 activity in cells synchronized in mitosis. In both cases, the abnormal mitosis induced by MK-1775 results in cell death in mitosis or cell death in interphase several hours following an extended mitosis. Paclitaxel also disrupts and induces a prolonged mitosis. Combining paclitaxel with low dose MK-1775 allows greater cell killing than either treatment alone in breast cancer cell lines.

## SUPPLEMENTARY MATERIALS FIGURES


