# Supplementary material for: Prolonged mitotic arrest induced by Wee1 inhibition sensitizes breast cancer cells to paclitaxel
Source: Oncotarget. 2017 May 13;8(43):73705–22. doi: 10.18632/oncotarget.17848 (PMC5650293; doi:10.18632/oncotarget.17848)
Supplement: Supplementary file 1 [file oncotarget-08-73705-s001.pdf]

Prolonged mitotic arrest induced by Wee1 inhibition sensitizes breast cancer cells to paclitaxel

Supplementary Materials

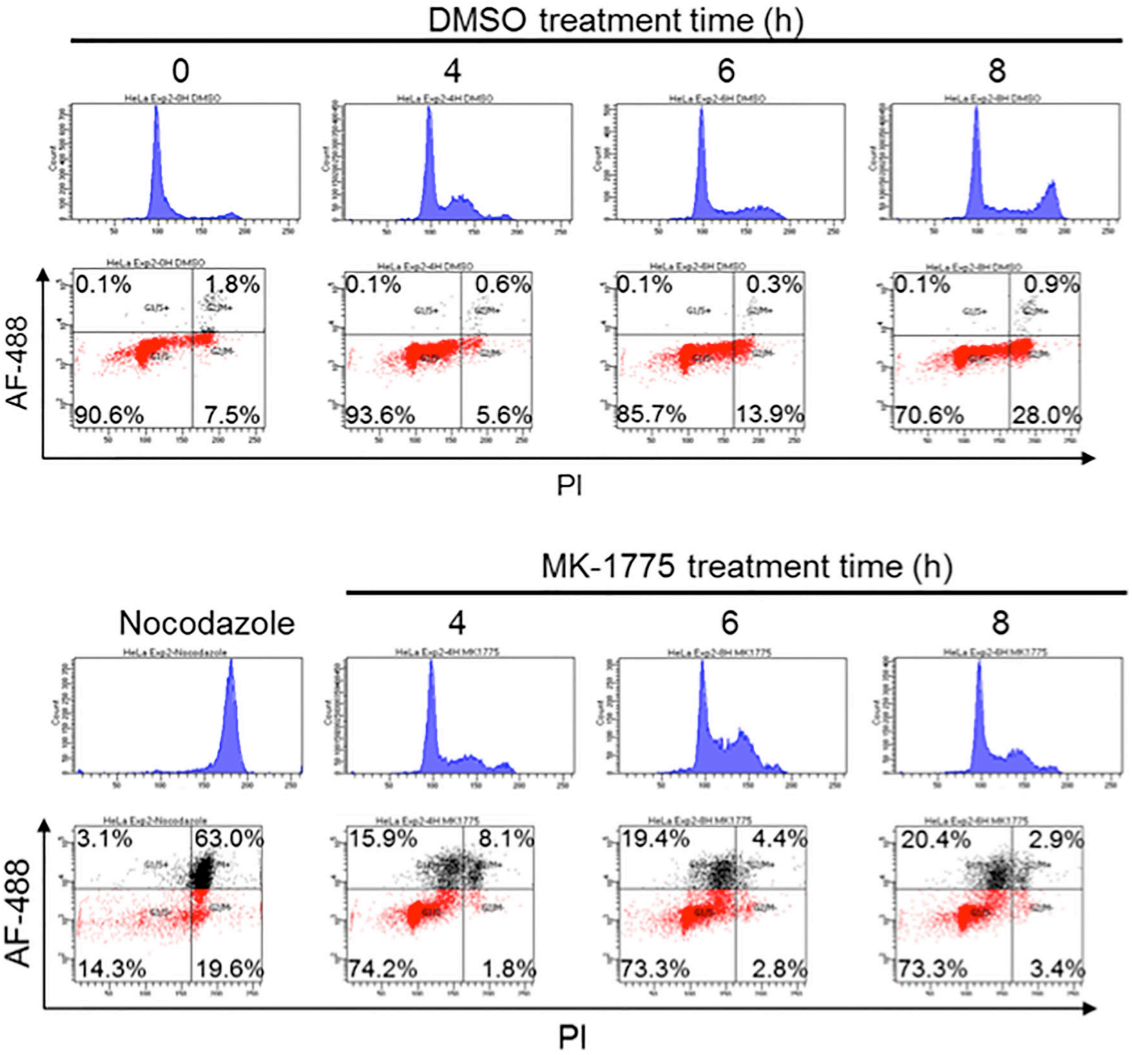

Supplementary Figure 1: MK-1775 induces premature mitosis in G1/S synchronized cells. HeLa cells were synchronized in G1/S phase by double thymidine block and then released into media containing with DMSO or MK-1775. Cells were fixed at indicated times, then stained for phospho-Ser10-histone H3 (PH3) and DNA, and analyzed by FACS to determined cell cycle phase. FACS analysis of nocodazole treated cells on the bottom left was mitotic control Histogram of DNA content is shown for indicated treatments at each time. Scatterplots show cells positive for PH3 (Black = positive; Red = negative). Right side of each dot blot represents cells in G2/M whereas left side represents cells in G1/S. Data are plotted in Figure 1D.

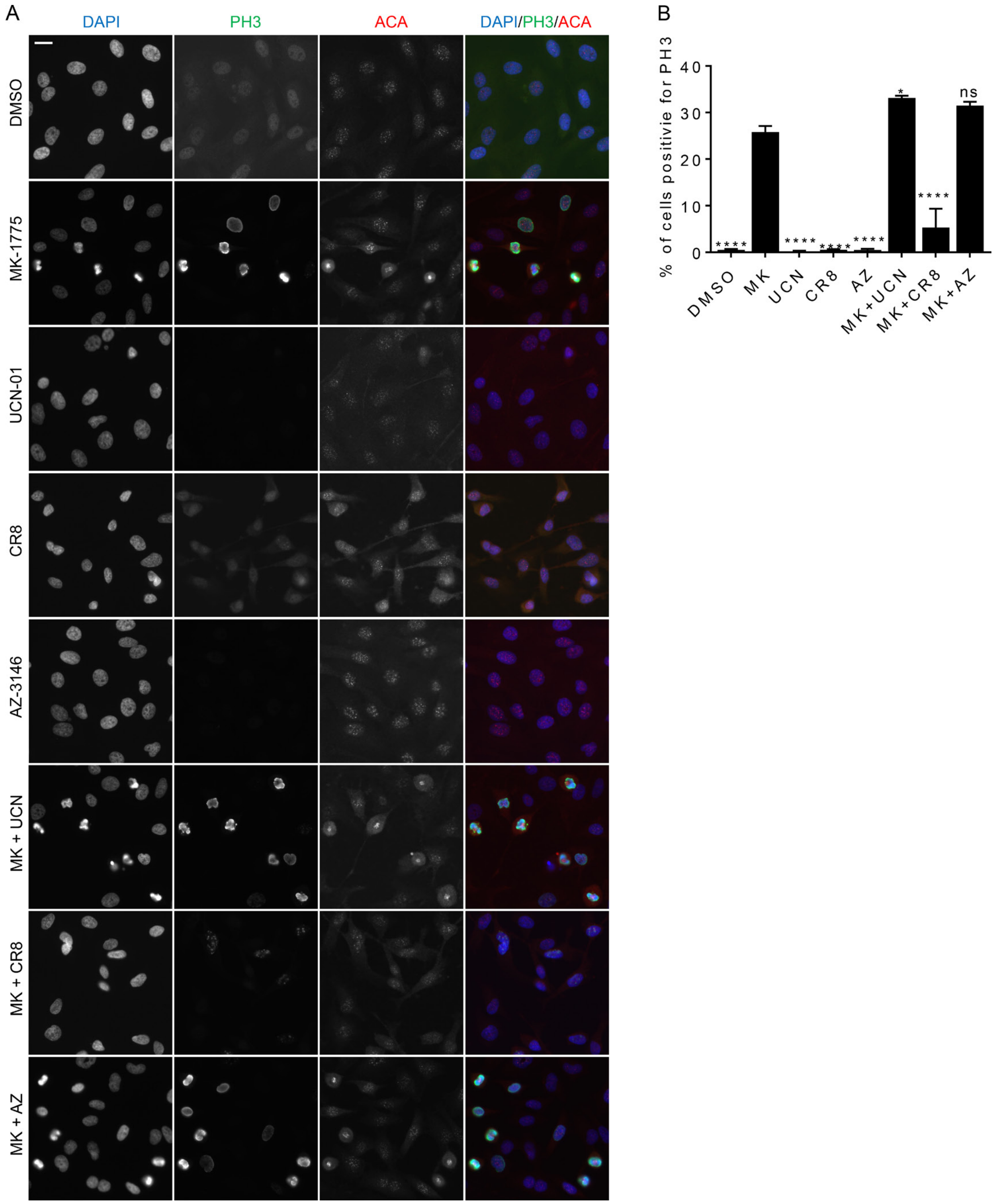

Supplementary Figure 2: Co-inhibition of Wee1 and Chk1 increased premature entry into mitosis. (**A**) HeLa cells were released from G1/S phase and then treated with different kinase inhibitors (Chk1 inhibitor UCN-01 (UCN), Cdk1 inhibitor CR8, and Mps1 inhibitor AZ-3146 (AZ)) alone or in the presence of MK-1775. MK-1775 alone and DMSO were included as controls. After a 4 h treatment, cells were stained for DNA (DAPI), centromere (ACA), and PH3 and analyzed by immunofluorescence microscopy. Scale bar = 20 µM. (**B**) The average percentage of cells positive for PH3 relative to DNA staining is shown for each treatment. Error bars represent standard error of the mean. Experiment was repeated three times. One-way ANOVA and Dunnett's multiple comparisons test (MK verses treatment) were used to determine sigificance level (*$p < 0.05$; ****$p < 0.0001$).

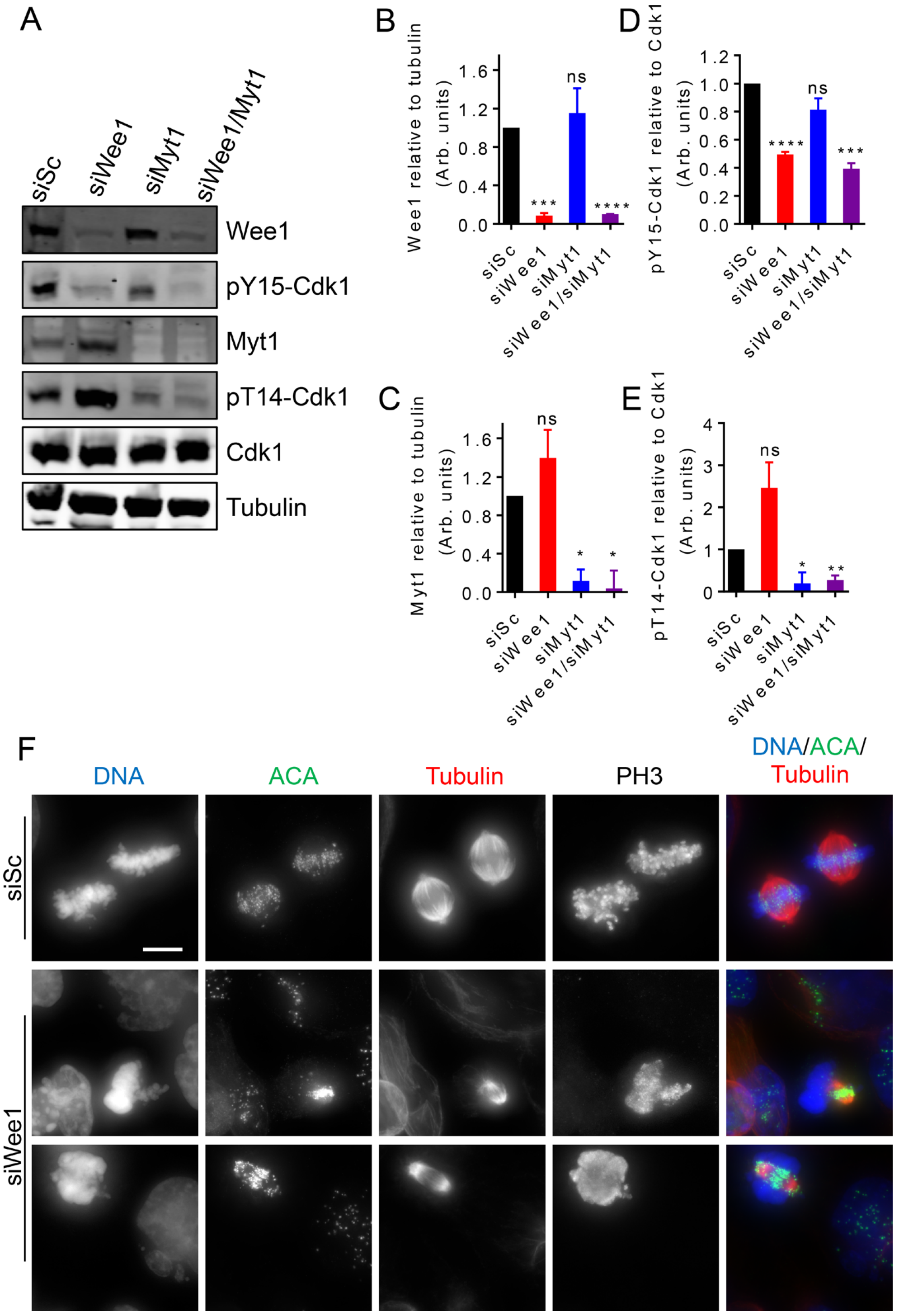

Supplementary Figure 3: siRNA mediated knockdown of Wee1 induces centromere fragmentation. HeLa cell extracts were prepared following transfections with scrambled siRNA or siRNAs against Wee1, Myt1, or both Wee1 and Myt1. (**A**) Extracts were analyzed by western blot to detect levels of Wee1, Myt1, pY15-Cdk1, pT14-Cdk1, Cdk1, and tubulin. Average levels of (**B**) Wee1 and (**C**) Myt1 relative to tubulin and (**D**) pY15-Cdk1 and (**E**) pT14-Cdk1 relative to Cdk1 are plotted. Error bars represent standard error of the mean. Student *t*-test were used to determine significance (*$p < 0.05$, **$p < 0.005$, ***$p < 0.0005$ and ****$p < 0.0001$) (**F**) Mitotic cells post transfection with scrambled siRNA or siRNA against Wee1 were fixed and then stained for histone H3 phospho-Ser10 (PH3), tubulin, anti-centromere antibody (ACA), and DNA. Experiments were repeated at least three times.

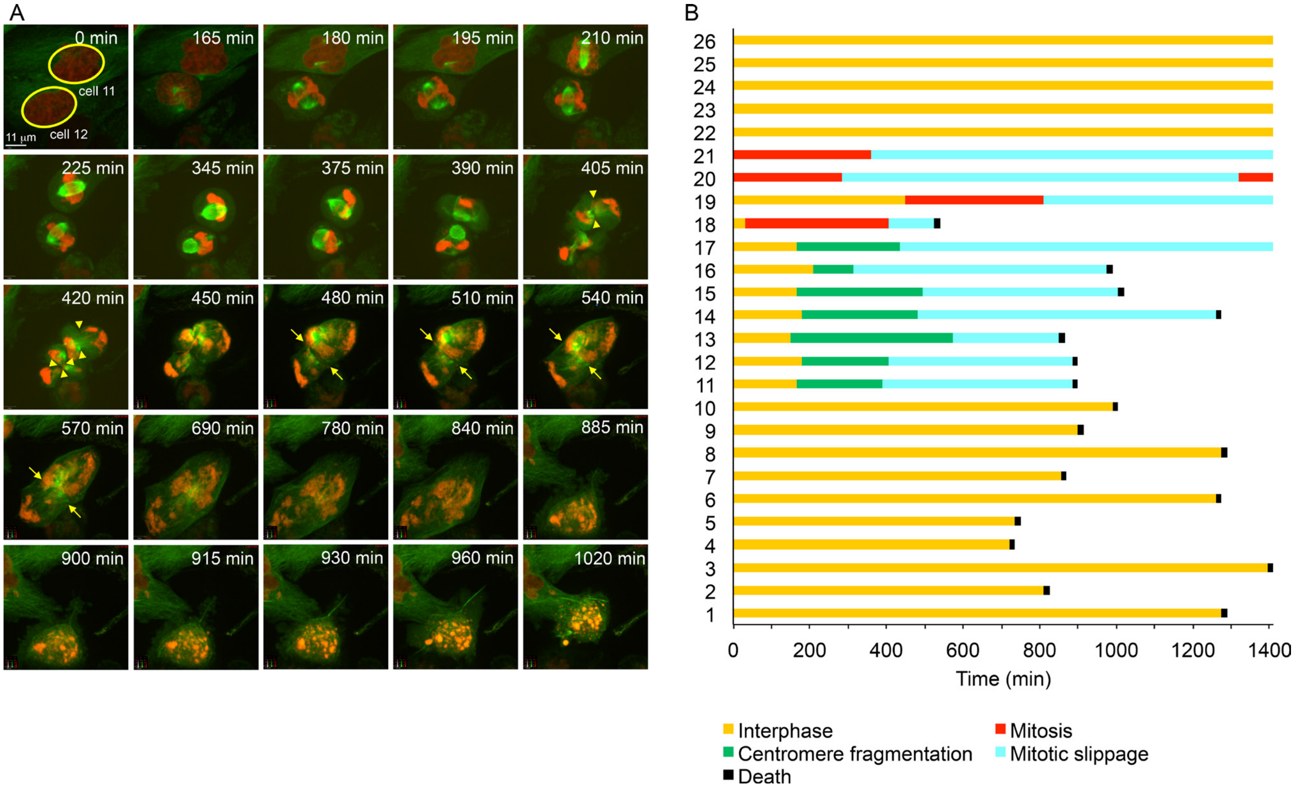

Supplementary Figure 4: Inhibition of Wee1 kinase induces prolonged mitosis and cell death. (**A**) Time-lapse panels of movie showing cells 11 and 12 as an example of the prolonged mitotic arrest and cell death phenotype. Red – mCherry histone H2B; green – EGFP-tubulin; yellow arrow heads indicate planes of cleavage of attempted anaphase; yellow arrows indicate plane of fusion of cells 11 and 12 at approximately 540 min of the movies; scale bar = 11 µm. Time is duration from the start of the time-lapse movie. Cell 12 is the same cell depicted in Fig 3B lower panel. (**B**) 26 individual HeLa cells stably expressing mCherry-H2B and EGFP-tubulin were synchronized in G1/S phase and then released into fresh media containing MK-1775. Duration of each indicated events was measured by time-lapse microscopy.

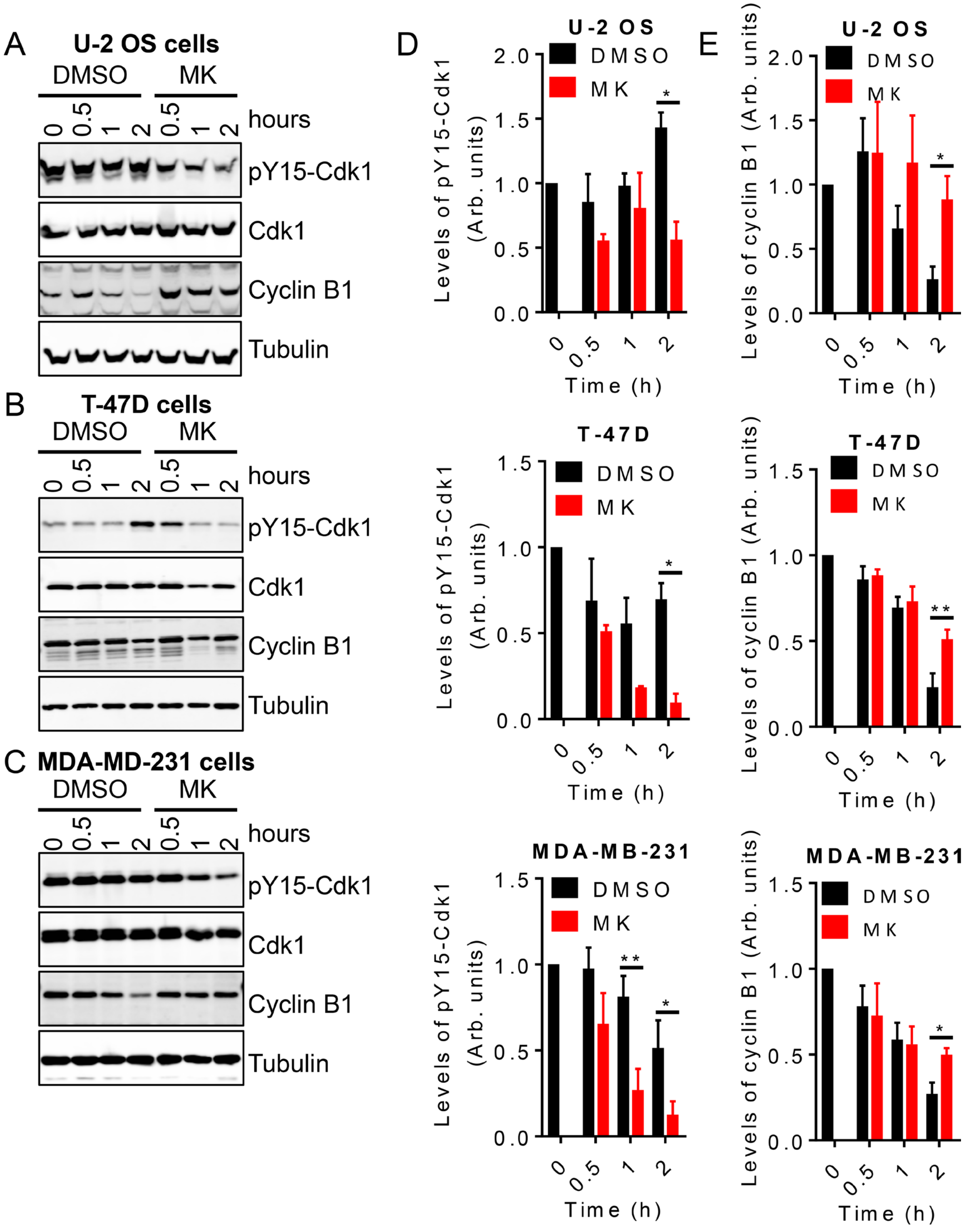

Supplementary Figure 5: Inhibition of Wee1 prevents normal mitotic exit. (**A**) U-2 O S, (**B**) T47-D, and (**C**) MDA-MD-231 cell lines were synchronized in prometaphase and then released into media containing either DMSO or MK-1775 (MK) for up to two hours. Cell extracts were prepared at indicated times. Extracts were analyzed by western blot for the levels of pY15-Cdk1, Cdk1, cyclin B1, and tubulin. (**D**) Average levels of pY15-Cdk1 (relative to Cdk1) and (**E**) cyclin B1 (relative to tubulin) were measured over time. Levels of pY15-Cdk1 and Cyclin B1 at time zero were set as 1. Error bars represent standard error of the mean. Statistical significance was determined using student *t*-test (*$p < 0.05$ and **$p < 0.005$). Experiments were repeated at least three times.

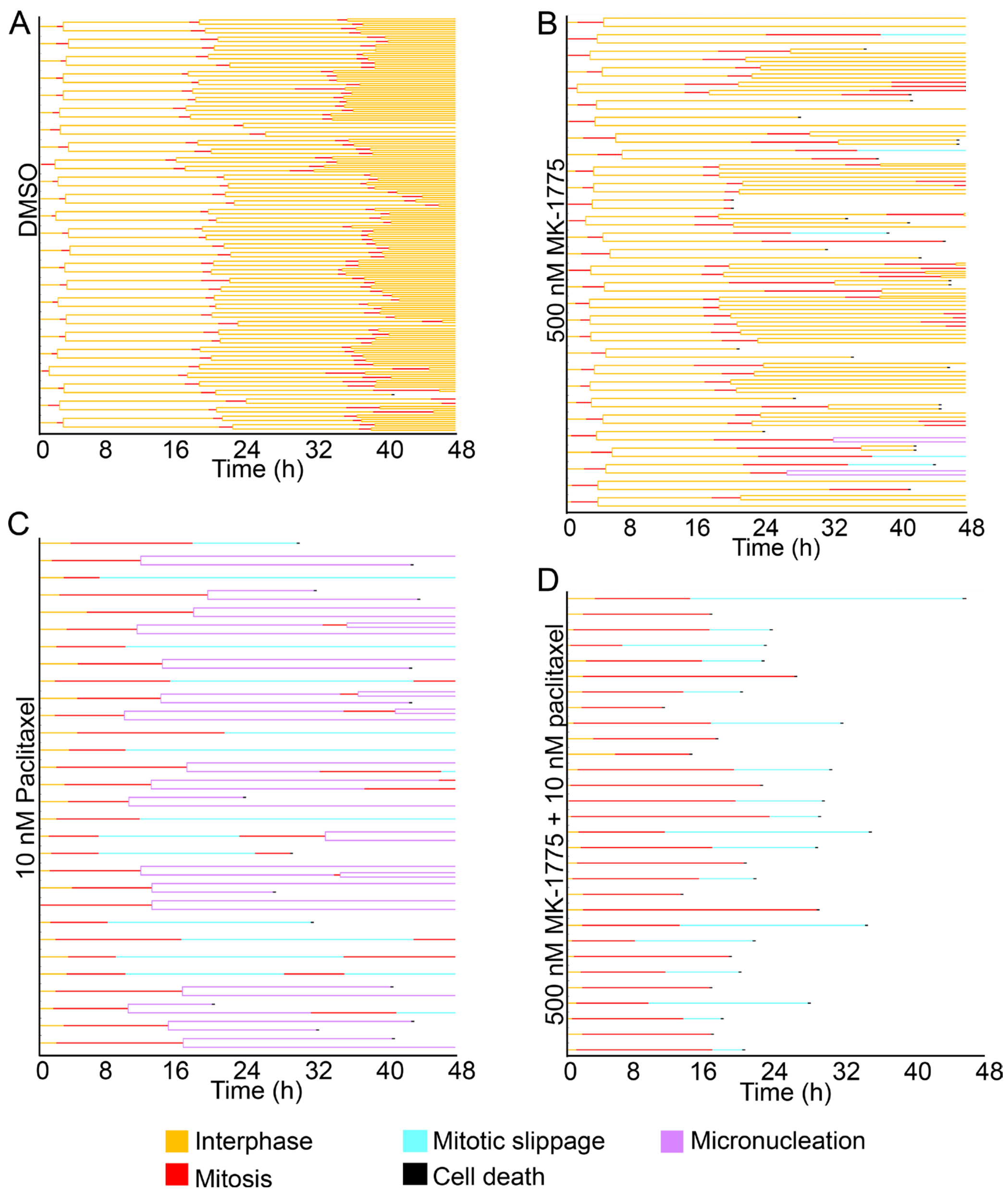

Supplementary Figure 6: Paclitaxel and MK-1775 co-treatment prevents mitotic exit. HeLa cells stably expressing EGFP-H2B were released from G1/S phase for 9 hours and then treated with (**A**) DMSO, (**B**) 1 mM MK-1775, (**C**) 10 nM paclitaxel, or (**D**) both 1 mM MK-1775 and 10 nM paclitaxel for 48 h. A line graph for individual mitotic cells tracked by time-lapse microscopy is shown, which includes times for indicated cellular events. A fork in the line indicates cell division and cell fate of daughter cells is also shown. Duration of mitotis (NEBD to anaphase) for indicated treatments are plotted in Figure 6A.
